# Seroprevalence and characterization of *Brucella* species in cattle slaughtered at Gauteng abattoirs, South Africa

**DOI:** 10.1002/vms3.190

**Published:** 2019-08-14

**Authors:** Francis B. Kolo, Abiodun A. Adesiyun, Folorunso O. Fasina, Charles T. Katsande, Banenat B. Dogonyaro, Andrew Potts, Itumeleng Matle, Awoke K. Gelaw, Henriette van Heerden

**Affiliations:** ^1^ Department of Veterinary Tropical Diseases University of Pretoria Pretoria South Africa; ^2^ Department of Production Animal Studies University of Pretoria Pretoria South Africa; ^3^ Department of Basic Veterinary Sciences Faculty of Medical Sciences University of the West Indies St. Augustine Trinidad and Tobago; ^4^ Gauteng Department of Agriculture and Rural Development Johannesburg South Africa; ^5^ Agricultural Research Council - Onderstepoort Veterinary Research Pretoria South Africa

**Keywords:** abattoirs, *Brucella abortus*, *Brucella melitensis*, Brucellosis, cattle, seroprevalence

## Abstract

**Background:**

Brucellosis is an infectious and contagious zoonotic bacterial disease of both humans and animals. In developing countries where brucellosis is endemic, baseline data on the prevalence of brucellosis, using abattoir facilities, is important.

**Objectives:**

The aim of this study was to determine the seroprevalence of antibodies against *Brucella* in slaughter cattle at Gauteng province, South Africa and to characterize isolates of *Brucella* spp.

**Methods:**

In this cross‐sectional study, un‐clotted blood samples with corresponding organ tissue samples were collected from slaughtered cattle. Serological [Rose Bengal test (RBT), complement fixation test (CFT) and indirect ELISA (iELISA)], molecular (PCR) and bacteriological methods were used to detect *Brucella* antibodies and *Brucella* spp. from 200 slaughtered cattle in 14 abattoirs.

**Results:**

The RBT revealed a seroprevalence of brucellosis as 11.0% (22 of 200) and iELISA confirmed 5.5% (11 of 200). The estimated seroprevalence from RBT and iELISA was 5.5% while RBT and CFT was 2.0% (4 of 200). *Brucella melitensis* (*n* = 6) and *B. abortus* (*n* = 5) were isolated from 11 cattle tissues (5.5%) as confirmed to species level with AMOS PCR and differentiated from vaccine strains with Bruce‐ladder PCR. Seven of the 11 isolates originated from seropositive cattle of which five were biotyped as *B*. *abortus* bv 1 (*n* = 2) and *B. melitensis bv* 2 (*n* = 1) and *B. melitensis* bv 3 (*n* = 2).

**Conclusions:**

This is the first documentation of *B. melitensis* in cattle in South Africa. The zoonotic risk of brucellosis posed by *Brucella*‐infected slaughter cattle to abattoir workers and consumers of improperly cooked beef cannot be ignored.

## Abstract 2

Seroprevalence and characterization of brucellosis in cattle slaughtered at Gauteng abattoirs, South Africa.

## Graphical abstract

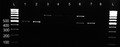

## 1 INTRODUCTION

Brucellosis is an infectious and contagious zoonotic bacterial disease of humans and a wide range of domestic animals and wildlife, particularly the ruminants (Corbel, 2006; Radostits, Gay, Hinchcliff, & Constable, 2006; Smirnova et al., 2013) and some marine animals (Foster, Osterman, Godfroid, Jacques, & Cloeckaert, 2007; Scholz & Vergnaud, 2013). The *Brucella* species are Gram‐negative, non‐capsulated, facultative intracellular, non‐spore forming, cocco‐bacilli bacteria (Godfroid, 2012; Seleem, Boyle, & Sriranganathan, 2010; Smirnova et al., 2013). *Brucella* spp. infecting farm animals include *B*. *abortus, B. melitensis, B. suis* and *B. ovis* (Godfroid, Nielsen, & Saegerman, 2010; Smirnova et al., 2013). The *Brucella* species are known to have host preferences, although there could be cross‐infection with other hosts. *Brucella abortus* has a host preference for the cattle but it can cause infection in other hosts including humans (undulant fever). *Brucella melitensis*, has a host preference for sheep and goats and it is the most pathogenic of the *Brucella* spp. that causes infection in humans (Malta fever). *Brucella canis* and *B. suis* has host preferences for dogs and pigs, respectively, and can cause brucellosis in humans (Alton, 1990; Carmichael, 1990; Godfroid et al., 2005; Pappas, 2013). Brucellosis has been eradicated or well controlled in developed countries (Pappas, Papadimitriou, Akritidis, Christou, & Tsianos, 2006). However, in many of the low and middle income countries (LMICs) such as in Africa, South and Central America, Middle East, Asia, Mediterranean Basin and the Caribbean, brucellosis is still common and high in occurrence both in the animal and human populations (Adesiyun & Cazabon, 1996; Godfroid et al., 2005; Olsen & Palmer, 2014; Pappas et al., 2006). Brucellosis may have existed in South Africa as an ancient disease as suggested by a paleopathological analysis study on the fossil of the late *Pliocene hominin* species (D'Anastasio, Zipfel, Moggi‐Cecchi, Stanyon, & Capasso, 2009). It had been suspected in 1898 that goats may have been the source of suspected cases of undulant fever which was also called “camp fever” in 40 patients around the Kimberley area of South Africa, an area where diamond mines were operating (Strachan, 1932; Van Drimmelen, 1949). Serological diagnosis, as well as cultural isolation, which identified *B. melitensis*, were relied upon for diagnosis from 1902 to 1911 (Strachan, 1932; Zammit, 1905) from samples including human blood, goat serum and milk samples in South Africa (Strachan, 1932). *Brucella melitensis* outbreaks have been documented in sheep in 1965 (Van Drimmelen, 1965), in goats in 1989 that was identified as *B. melitensis* bv 1 (Ribeiro, Herr, & Chaparro, 1990), 1994 (Reichel, Nel, Emslie, & Bishop, 1996), 2000 (Emslie & Nel, 2002), 2007 and 2015 (DAFF, 2015). *Brucella abortus* infection in South Africa was reported in 1913, when contagious abortion was observed to spread across the country in cattle (Van Drimmelen, 1949). In South Africa, *B. abortus* bv 1 predominantly cause infection in cattle (90%) and *B*. *abortus* bv 2 to a lesser extent (Godfroid, Bishop, Bosman & Herr, 2004). Mauff (1980) reported fives cases of brucellosis associated with by‐products (condemned meat and unborn calves) at a new abattoir in South Africa. These were confirmed cases based on serological tests and culture and it was reported that the affected individuals did not wear any protective clothing at the by‐product facility (Mauff, 1980). Despite this risk and threat of exposure of humans to brucellosis at abattoirs, the only published report of an abattoir‐based study on bovine brucellosis was in 1984 where a prevalence rate of 1.5% was reported for cattle sampled at Cato Ridge abattoir in KwaZulu‐Natal province (Bishop, 1984). In the Eastern Cape province, a 9.2% prevalence rate of *B. abortus* (of which 0.8% *B. abortus* S19 vaccine strain) was isolated from cattle, 2.9% *B. melitensis* from sheep and 6.3% *B. melitensis* from goats using different samples (blood, milk and lymph nodes) followed by species specific confirmation using PCR (Caine, Nwodo, Okoh, & Green, 2017).

In South Africa, brucellosis is a reportable disease. Control measures have been instituted to prevent the spread of brucellosis in the country with the focus mainly on bovine brucellosis through the animal diseases Act 35 of 1984 and the bovine brucellosis scheme (R.2483 of 9 Dec 1988) which is regulated by the Director of Animal Health at the Department of Agriculture, Forestry and Fisheries (DAFF). Currently the testing scheme for bovine brucellosis (established under section 10 of the Animal Disease act 35) is compulsory for only high‐risk herds that have been confirmed or suspected of infection using Rose Bengal test (RBT) and the complement fixation test (CFT). Entering the brucellosis testing scheme is voluntary for all other bovine herds and livestock owners. Vaccination is practised in South Africa according to the stipulated standards with mainly *B. abortus* S19 and to a lesser extent *B. abortus* RB 51 in cattle while *B. melitensis* Rev 1 is used in sheep and goats (OIE, 2016). These control measures amongst others are instituted to prevent a spillover of the disease to other domestic animals and wildlife in areas close to the wildlife parks (Simpson et al., 2018).

In South Africa, an estimation of over 3,476,000 of cattle were slaughtered from September 2015 to August 2016 in the abattoirs (DAFF, 2016). These abattoir facilities can also be used to monitor disease control policies, detect newly introduced disease agents and to assess intervention programmes, such as brucellosis vaccination, and most importantly, abattoir survey may also facilitate early intervention to mitigate the epidemic loss of animals (Alton, Pearl, Bateman, McNab, & Berke, 2015; Fasina et al., 2015; Kaneene, Miller, & Meyer, 2006). As such, an abattoir surveillance study on brucellosis can generate baseline data on the occurrence of the disease among the animal population, especially when the animals come from various farms to be processed into wholesome meat products for human consumption (Alton et al., 2015; Fasina et al., 2015; Kaneene et al., 2006). However, abattoir data may not produce reliable prevalence estimates because the population of slaughtered cattle tends not to correctly represent the target population which may affect the validity of results from such facilities. Animals and carcasses of *Brucella*‐infected slaughtered animals can be a source of infection to susceptible abattoir workers, as these workers may be exposed to infection through direct contact with infected animal's secretions or blood, or indirectly through the consumption of raw meat or undercooked meat (Corbel, 2006). It has been documented in the literature that the risk of carcass contamination by bacteria, including *Brucella* spp., increases with the throughput, i.e. the number of animals slaughtered (Sadler, 1960). Due to the predominant voluntary nature of the brucellosis testing scheme in South Africa, known and unknown brucellosis infected cattle can be slaughtered at abattoirs. The aim of this study was to determine the seroprevalence of antibodies against *Brucella* and characterize *Brucella* spp. from slaughtered cattle from abattoirs.

## 2 MATERIALS AND METHODS

### 2.1 Study area, study design and sample size

A cross‐sectional study was conducted to determine the seroprevalence of brucellosis and to detect, isolate and characterize *Brucella* spp. in cattle slaughtered at the Gauteng province abattoirs from April 2016 to April 2017.

The study area was the Gauteng Province of South Africa. The Province is the smallest province in South Africa with approximately 1.5% (surface area of 1,219,602 km^2^) of the land area, yet it remains most populated, accounting for approximately 23.7% of the country's population. Although the most recent estimated number of cattle in the Gauteng province in May 2018 was 246,395, it is known that a large number of cattle from other provinces move to Gauteng on regular basis (DAFF, 2018)**.**

**Figure 1:**
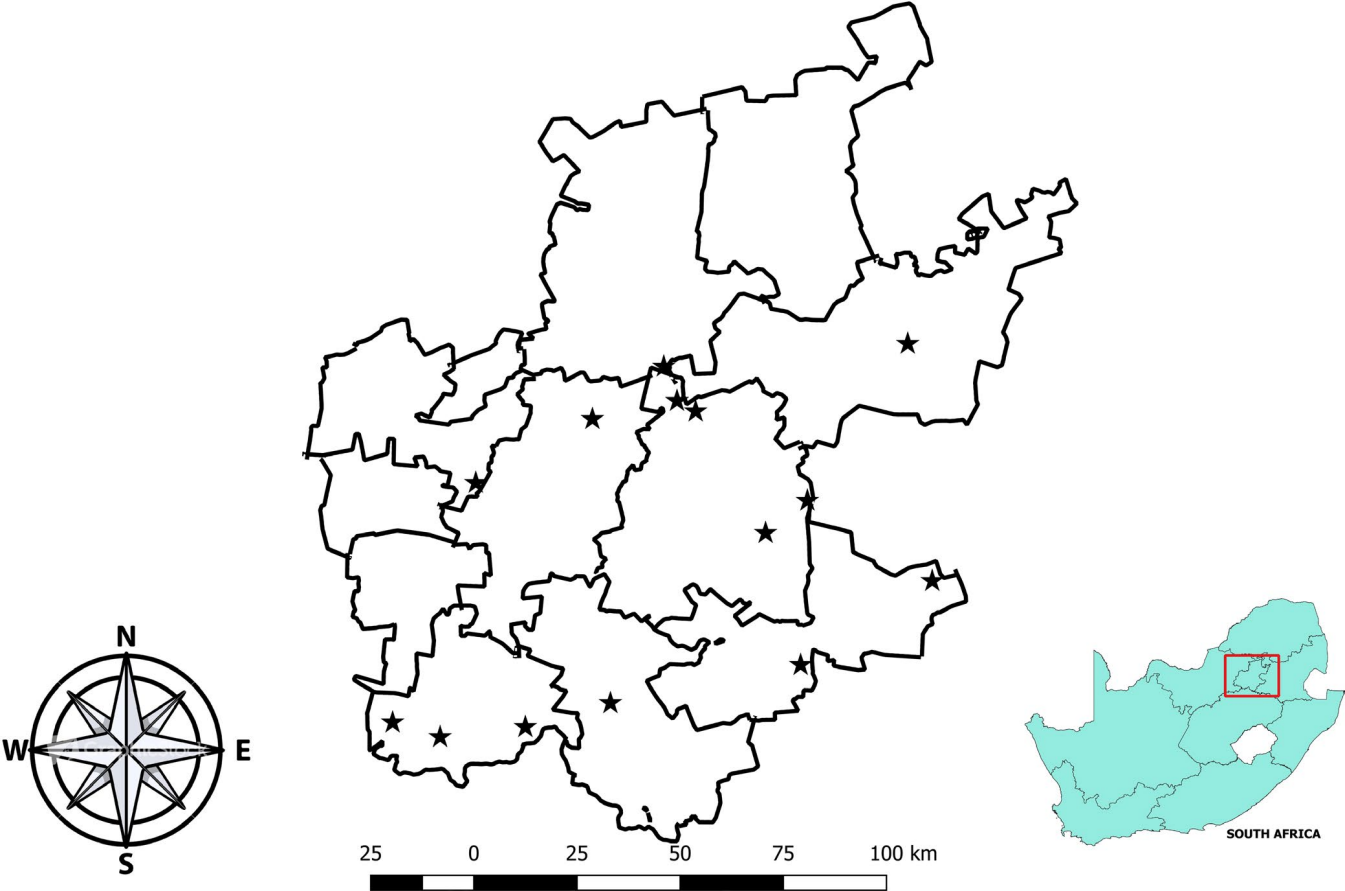
Locations of abattoirs sampled in the Gauteng Province of South Africa

Fourteen abattoirs in the Gauteng Province (Figure 1), which were operational and consented to participate in the study, were randomly selected from 28 operational abattoirs identified. These abattoirs were categorized into high throughput (*n* = 7) slaughtering more than 20 cattle per day and low throughput (*n* = 7) where 20 or less cattle are slaughtered daily. Among the selected abattoirs for the study, 13 operated as multi‐species and 1 was mono‐species facilities.

### 2.2 Sample size and collection

In this cross‐sectional study, a systematic simple random sampling method was used to determine the sample size and power. Sample size was estimated at 50.0% expected frequency, with 5.0% acceptable margin of error and a design effect of 1 and clusters equals to 1 in Epi‐Info 7 version 10. At a 95.0% confidence level, the sample size of 200 animals was achieved. A systematic sampling method was used at selected abattoirs. For all selected animals, blood samples were collected as follows:

Unclotted blood was collected from 200 cattle at the point of slaughter using sterile 50 ml cups with approximately 5 ml of the blood aliquoted into yellow‐capped vacutainer tubes. Corresponding tissue samples comprising of lymph nodes, spleen and liver were collected from each of the cattle. The lymph nodes of each animal were pooled and comprised the retropharyngeal, parotid, submandibular and mesenteric lymph nodes. The excised tissues were processed according to set laboratory protocols in a bio‐safety laboratory level 2 hood. The homogenized tissues were used for DNA extraction (PCR) and bacterial isolation. Demographic data comprising of the animal species, breed, sex, age, the farm origin and the identity of the abattoir where the animal was slaughtered were collected for each of the animals slaughtered. The age of the animals was determined using the dental formula as described by Eubanks (2012). The vaccination status of the animals could not be ascertained.

### 2.3 Serological test methods on serum samples

Serological procedures were carried out using three different procedures including the RBT, the CFT and the indirect enzyme‐linked immunosorbent assay (iELISA). For this study, because RBT is unable to differentiate between antibodies produced in response to vaccination or natural exposure, its results were not used as stand‐alone percentage estimates but only as part of a serial test in a combination with either CFT or iELISA results.

Rose Bengal test was conducted as described by OIE (2009). The diagnostic sensitivity and specificity for the RBT have earlier been confirmed to be 100.0% and 75.0%, respectively, based on previous validation studies (Nielsen et al., 2005; Stemshorn et al., 1985).

CFT was performed on the cattle sera at the Agricultural Research Council‐ Onderstepoort Veterinary Research (ARC‐OVR) laboratory, South Africa using the OIE protocol (OIE, 2009). The cut‐off value for this test was ≥30 IU/ml as an indication of infection and the obtained values were compared with the positive and negative controls. The IDEXX brucellosis serum ×2 ELISA Test kit from Pourquier^®^, IDEXX, Switzerland were used according to the manufacturer's instruction. The cut‐off value for determination of antibody‐positive status in cattle recommended by IDEXX is 80.0%.

### 2.4 Bacterial isolation from tissue samples

Homogenate (200 μl) from each tissue (lymph nodes, spleen and liver) was inoculated onto Farrell's and modified CITA media, respectively, and incubated at 37°C with 5.0% CO_2_. Plates were observed for bacterial colony growth for 10 days. *Brucella* organisms were identified presumptively by morphology using the Stamp's modified Ziehl‐Neelsen staining method (OIE, 2009). Morphologically identified *Brucella* colonies were purified by sub‐culturing on the sheep blood agar media. Mixed or contaminated cultures were subjected to serial dilution 1:1,000 using buffered peptone water and inoculated onto the selective media until purified colonies were obtained. Pure *Brucella* cultures were biotyped at ARC‐OVR, South Africa according to the methods described by Ribeiro and Herr (1990).

### 2.5 Molecular detection

Genomic DNA was extracted from cultures of each homogenized tissue (lymph nodes, spleen and liver) and thereafter, the cells of *Brucella* cultures were purified using the set protocol according to Isolate II Genomic DNA kit by Bioline (South Africa). Detection of DNA and isolation procedure for *Brucella* spp. isolation was performed on all animals, regardless of their serological status. Genus‐specific 16S‐23S rRNA interspacer region (ITS) PCR was used to amplify *Brucella* region and *B*. *abortus* strain 544 and *B. melitensis* Rev 1 served as positive controls as described by Keid et al. (2007) for detection *Brucella* DNA in tissue samples (lymph nodes, spleen and liver). The process amplified a 214 bp fragment using primers (ITS66: ACATAGATCGCAGGCCAGTCA and ITS279: AGATACCGACGCAAACGCTAC). Primers were used at a final concentration of 0.2 μM with 1× DreamTaq Green PCR Master Mix (ThermoFisher Scientific, South Africa) and 2 μl DNA in a 15 μl PCR reaction. The PCR cycling condition consisted of 95°C for 3 min, followed by 35 cycles at 95°C for 1 min, 60°C for 2 min, 72°C for 2 min and a final extension at 72°C for 5 min. PCR products were analysed by electrophoresis using a 2.0% agarose gel stained with ethidium bromide and viewed under UV light.

The multiplex AMOS PCR assay that identifies and differentiates *B. abortus*,* B. melitensis*,* B. ovis* and *B. suis* was conducted as described (Bricker & Halling, 1994; Weiner, Iwaniak, & Szulowski, 2011) using DNA extraction from cultures. Four species‐specific forward primers were used at a final concentration of 0.1 μM with 0.2 μM reverse primer *IS711* (Table 1) with 1× MyTaq™ Red PCR Mix (Bioline, South Africa) and 2 μl of template DNA in 25 μl PCR reaction. PCR cycling condition was initial denaturation at 95°C for 5 min followed by 35 cycles at 95°C for 1 min, 55.5°C for 2 min, 72°C for 2 min and a final extension step at 72°C for 10 min. PCR products were analysed by electrophoresis using a 2.0% agarose gel stained with ethidium bromide and viewed under UV light.

**Table 1:** Sequences and characteristics of the oligonucleotide primers used for different *Brucella* species in the AMOS PCR assay

PCR name	Primer name	Sequence (5′‐ 3′)	DNA Targets	Amplicon (bp)	Concentration (μM)	Reference
AMOS	*B. abortus*	GAC GAA CGG AAT TTT TCC AAT CCC	*IS711*	498	0.1	(Bricker, Ewalt, Olsen, & Jensen, 2003; Bricker & Halling, 1994)
	*B. melitensis*	AAA TCG CGT CCT TGC TGG TCT GA		731	0.1	
	*B. ovis*	CGG GTT CTG GCA CCA TCG TCG GG		976	0.1	
	*B. suis*	GCG CGG TTT TCT GAA GGT GGT TCA		285	0.1	
	*IS711*	TGC CGA TCA CTT AAG GGC CTT CAT			0.2	

A multiplex Bruce‐ladder PCR assay to identify and differentiate between vaccine strains and field isolates of *Brucella* spp. was conducted as described (García‐Yoldi et al., 2006; Lopez‐Goñi et al., 2008; Weiner et al., 2011) (Table 2). Eight species‐specific forward and reverse primers were used at a final concentration of 6.25 μM with 1× MyTaq™ Red PCR Mix (Bioline, South Africa) and 2 μl of template DNA in a 25 μl PCR reaction. The PCR cycling condition included an initial denaturation cycle at 95°C for 5 min followed by 25 cycles at 95°C for 30 s, at 64°C for 45 s, and at 72°C for 3 min and a final extension step at 72°C for 10 min. PCR products were analysed by electrophoresis using a 2.0% agarose gel stained with ethidium bromide and viewed under UV light.

**Table 2:** Sequences and characteristics of the Bruce‐ladder PCR assay primers used in the study

PCR name	Primer name	Sequence (5′‐ 3′)	DNA targets	Amplicon (bp)	Concentration (μM)	Reference
Bruce‐ladder	BMEI0998f	ATC CTA TTG CCC CGA TAA GG	*wboA*	1682	6.25	(Lopez‐Goñi et al., 2008)
	BMEI0997r	GCT TCG CAT TTT CAC TGT AGC				
	BMEI0535f	GCG CAT TCT TCG GTT ATG AA	*bp26*	450	6.25	
	BMEI0536r	CGC AGG CGA AAA CAG CTA TAA				
	BMEII0843f	TTT ACA CAG GCA ATC CAG CA	*omp31*	1071	6.25	
	BMEII0844r	GCG TCC AGT TGT TGT TGA TG				
	BMEI1436r	ACG CAG ACG ACC TTC GGT AT	Deacetylase	794	6.25	
	BMEI1435r	TTT ATC CAT CGC CCT GTC AC				
	BMEII0428f	GCC GCT ATT ATG TGG ACT GG	*eryC*	587	6.25	
	BMEII0428r	AAT GAC TTC ACG GTC GTTCG				
	BR0953f	GGA ACA CTA CGC CAC CTT GT	ABC	272	6.25	
	BR0953r	GAT GGA GCA AAC GCT GAA G	Transporter			
	BMEI0752f	CAG GCA AAC CCT CAG AAG C	*rpsL*	218	6.25	
	BMEI0752r	GAT GTG GTA ACG CAC ACC AA				
	BMEII0987f	CGC AGA CAG TGA CCA TCA AA	CRP	152	6.25	
	BMEII0987r	GTA TTC AGC CCC CGT TAC CT	Regulator			

### 2.6 Statistical analysis

Data collected were managed using the Microsoft Excel version 2007. The R software (RCoreTeam, 2013) was used to analyse the data and to conduct descriptive analysis, and Epi‐Info 7 version 10 was used to conduct analyses of frequency with 95% confidence interval, and calculated odds ratio and Chi‐Square tests to examine differences between groups.

Animal prevalence was determined by the number of positive animals divided by the total number of animals sampled.

### 2.7 Ethical approval

Ethics approval for the study was obtained from ARC‐OVI Animals Ethics Committee (AEC12‐16), University of Pretoria Animal ethics Committee (V089‐16). Section 20 approval was granted according to Act 35 of 1984 by the Directorate of Animal Health, South Africa.

## 3 RESULTS

Out of the cattle samples tested, 57.5% (115/200) were from high throughput (HT) abattoirs, and 42.5% (85/200) originated from low throughput (LT) abattoirs. Of the 200 heads of cattle, 41.0% (82/200) were female while 59.0% (118/200) were male. The distribution of the cattle stratified by age was 92.0% (184/200) for adult and 8.0% (16/200) for young cattle; the distribution stratified by breed was Bonsmara, 69.5% (139/200), Nguni, 16.5% (33/200), Brahman, 5.5% (11/200), Jersey, 5.0% (10/200) and Holstein, 3.5% (7/200).

Among the 200 cattle tested, 11.0% (22/200) were positive on RBT, 5.5% (11/200) were positive on iELISA while only four of the 22 (18.2%) RBT positive sera were confirmed positive on CFT (2.0%, 4/200). The estimated seroprevalence from RBT and confirmed by iELISA was 5.5%. While the seroprevalence from RBT confirmed with CFT was 2.0%. *Brucella* DNA detection rate from the screened cattle tissues by ITS‐PCR was 12.5% (25/200) (Table 3). *Brucella* spp. isolation rate was 5.5% (11/200).

**Table 3:** Prevalence and risk of *Brucella* spp. from abattoirs samples by seropositivity, 16S‐23S rRNA interspacer region (ITS) PCR and the isolation rate

Animal demography	Total	Serology positives †(%)	‡OR (95% § CI)	Chi square	*p*‐value
Seropositivity to *Brucella* spp. stratified by sex, age of animals and type of abattoirs sampled					
Sex		(RBT)			
Female	82	16 (19.5)	4.5 (1.67–12.13)	8.9	0.00
Male	118	6 (5.1)			
Sex		(ELISA)			
Female	74	8 (9.8)	0.2 (0.06–0.94)	3.5	0.05
Male	115	3 (2.5)			
Age		(RBT)			
Adult	184	18 (9.8)	0.3 (0.09–1.11)	2.1	0.15
Young	16	4 (25.0)			
Age		(ELISA)			
Adult	176	8 (4.4)	5.1 (1.20–21.5)	3.4	0.06
Young	13	3 (18.5)			
Animals positive by 16S‐23S rRNA interspacer region (ITS) ¶PCR stratified by sex, age and type of abattoirs sampled					
Sex					
Female	82	17 (20.7)	3.60 (1.47–8.80)	7.4	0.01
Male	118	8 (6.8)			
Age					
Adult	184	22 (12.0)	0.59 (0.15–2.23)	0.2	0.69
Young	16	3 (18.8)			
Abattoir type					
High throughput	115	6 (5.2)	0.19 (0.07–0.51)	11.4	0.00
Low throughput	85	19 (22.4)			
Isolation rate by bacteriological method stratified by sex, age of animals and type of abattoirs sampled					
Sex					
Female	82	8 (9.8)	4.14 (1.06–16.12)	3.6	0.06
Male	118	3 (2.5)			
Age					
Adult	184	9 (4.9)	0.36 (0.7–1.83)	0.5	0.48
Young	16	2 (12.2)			
Abattoir type					
High throughput	115	1 (0.9)	0.06 (0.01–0.52)	9.2	0.00
Low throughput	85	10 (11.7)			

† = percentage, ‡ = odds ratio, § = confidence interval, ¶ = polymerase chain reaction.

Of the 25 ITS‐PCR positives, 56.0% (14/25) were from seropositive cattle (RBT or ELISA), while 44.0% (11/25) were from seronegative cattle. The serology positivity, PCR detection and isolation rates among the cattle population according to sex and age with the chi‐square measure of association is shown in Table 3. Type of abattoir is an indication for carcass contamination that can be detected by molecular and bacteriological methods.

The distribution of cattle that tested seropositive by RBT and confirmed with iELISA according to breed was as follows: Bonsmara, 3.6% (5/139); Nguni, 9.1% (3/33); Holstein, 28.6% (2/7); Brahman, 9.1% (1/11) and Jersey, 0.0% (0/10). The differences were not statistically significant (*p* > 0.05). With the use of the molecular method, the distribution of positivity according to the breed was as follows: Bonsmara 10.8% (15/139), Nguni 12.1% (4/33), Holstein 42.8% (3/7), Brahman 27.3% (3/11) and Jersey 0.0% (0/10). The differences were not statistically significant (*p* > 0.05). Of the cattle tested with bacteriological methods, the distribution of isolation positivity according to the breed of the animals was as follows: Bonsmara, 4.3% (6/139), Nguni 3.0% (1/33), Holstein 42.8% (3/7), Brahman 9.1% (1/11) and Jersey 0.0% (0/10). The differences were not statistically significant (*p* > 0.05).

Of the tissue samples from the 200 slaughtered cattle tested using the ITS‐PCR for the detection of *Brucella* DNA, the frequencies of detection were 11.5% (23/200, 95%CI = 7.43–16.6) from the lymph nodes, 7.5% (15/200, 95%CI = 4.26–12.1) from the spleen and 7.0% (14/200, 95%CI = 3.88–11.5) from the liver. The detection rates from the three tissue samples among the 25 ITS‐PCR‐positive cattle are lymph nodes 92.0% (23/25), 64.0% (16/25) and 56.0% (14/25) for the lymph nodes, spleen and liver, respectively.

Of the 25 ITS‐PCR positive cattle, the frequency of detection was 68.0% (17/25, 95%CI = 46.5–85.1) in the females and 32.0% (8/25, 95%CI = 14.9–53.5) in the males. Of the 25 ITS‐PCR positive cattle, the distribution by age classification was 88.0% (22/25, 95%CI = 68.8–97.4) for adults and 12.0% (3/25, 95%CI = 2.5–31.2) for young cattle; and by abattoir types was 24.0% (6/25, 95%CI = 9.36–45.1) for HT abattoirs and 76.0% (19/25, 95%CI = 54.9–90.6) for LT abattoir.

Among the 23, 15 and 14 ITS‐PCR‐positive lymph nodes, spleen and liver tissues, respectively, the frequency of isolation was 26.1% (6/23) from the lymph nodes, 40.0% (6/15) from the spleen and 42.8% (6/14) from the liver samples. Of the total 25 PCR‐positive cattle, the frequency of *Brucella* spp. isolation was 44.0% (11/25, 95%CI = 24.4–65.1). The isolation rate from all the three tissue samples among the 11 culture‐positive cattle was 26.1% (6/23) for each tissue.

**Figure 2:**
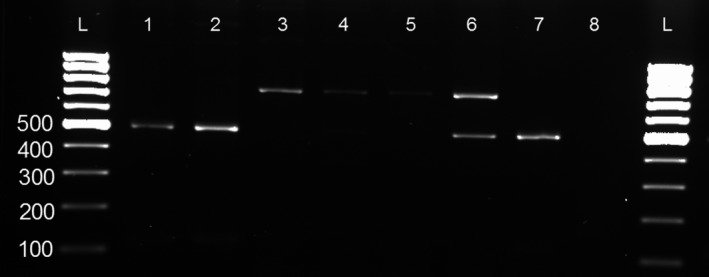
Gel electrophoresis of products from AMOS‐PCR of the *IS711* gene using species‐specific primers. Lanes 1 and 2 show amplification products of 498 bp in length for *Brucella abortus* and Lanes 3 to 6 show amplification products of 710 bp for *B. melitensis*, while Lanes 4 and 6 show double amplification as a result of mixed infection of both *B. abortus* and *B. melitensis*, in the smaples. Lanes 7 and 8 show the positive and negative control, lane L shows 100 bp DNA ladder (Invitrogen, ThermoFisher^®^ scientific, South Africa)

**Figure 3:**
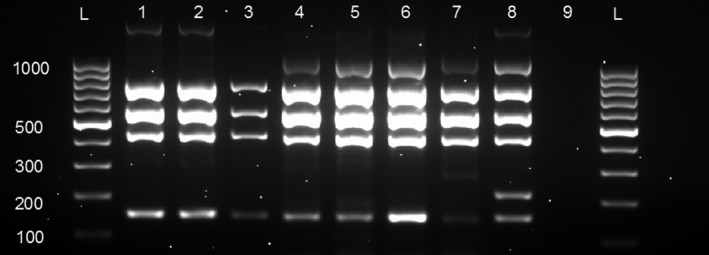
Gel electrophoresis of Bruce‐Ladder PCR amplification product using species specific primers. Lanes 1 and 2 show amplification product of *B. abortus,* lane 3 is *B. abortus* strain 544 (control), lanes 4 and 5 show amplification for *B. melitensis* (cattle) and lanes 6 and 7 show amplification for *B. melitensis* (sheep), lanes 8 and 9 show Rev 1 positive and negative controls, respectively, lane L shows 100 bp DNA ladder (Invitrogen, ThermoFisher^®^ scientific, South Africa)

Of the 11 confirmed *Brucella* isolates from cattle by the “gold standard” method of culture and isolation, 63.6% (7/11) were from the confirmed iELISA‐positive cattle. The other *Brucella* isolates were from RBT‐positive cattle (*n* = 3) except one from a seronegative cow. AMOS‐PCR assay characterized the seven isolates from the iELISA‐positives as *B*. *melitensis* (four isolates) and *B. abortus* (three isolates). The remaining four isolates, three of which were RBT‐positives, were classified as *B. melitensis* (two) and *B. abortus* (one); while the only single isolate that was negative on serology was characterized as *B. abortus*. In total, the AMOS‐PCR characterized the 11 isolates as six *B. melitensis* and five *B. abortus*. Two of these isolates had amplification for both *B. abortus* and *B. melitensis* (Figure 2). All isolates were differentiated from the vaccine strains using the Bruce‐ladder PCR assay (Figure 3).

From the 11 *Brucella* culture‐positive cattle, the frequency of isolation of *Brucella* spp. by sex was 72.7% (8/11, 95%CI = 39.0–94.0) in the females and 27.3% (3/11, 95%CI = 6.02–61.0) in the males and by age was 81.8% (9/11, 95%CI = 48.2–97.7) in adults and 18.2% (2/11, 95%CI = 2.28–51.8) in the young cattle. The frequency of isolation was 9.1% (1/11, 95%CI = 0.23–41.3) and 90.9% (10/11, 95%CI = 58.7–99.9) in the HT and LT abattoirs respectively.

Five of the 11 *Brucella* isolates were purified and biotyped. Two were determined to be *B. melitensis* bv 3, one as *B. melitensis* bv 2 and the remaining two as *B. abortus* bv 1. The five *Brucella* isolates were from iELISA‐positive cattle.

## 4 DISCUSSION

In this study, antibodies as well as the pathogen, were detected by a polyphasic approach using serology (RBT, CFT and iELISA), PCR and culturing. The brucellosis estimated seroprevalence and culture prevalence (gold standard) among the slaughter cattle at the Gauteng abattoirs is at 5.5%. *Brucella abortus* bv 1 was isolated in this study, which was expected since *B. abortus* bv 1 is the most dominant biovar causing infection in cattle (Coetzer, Thomson, & Tustin, 1994). For the first time *B. melitensis* bv 2 and 3 were also isolated from cattle tissue. In the past *B. melitensis* bv 1 has been isolated from goats (Reichel et al., 1996) and humans (Wojno et al., 2016), while *B. melitensis* bv 3 was isolated from sable in 2007 and 2015 as documented by DAFF (2015) and van Heerden (unpublished results).

As mentioned above, the testing scheme for bovine brucellosis (established under section 10 of the Animal Disease act 35) is compulsory for only high‐risk herds, while entering the brucellosis testing scheme is voluntary for all other bovine herds and livestock owners in South Africa. Due to the predominant voluntary nature of the brucellosis testing scheme in South Africa, known and unknown brucellosis infected cattle can be slaughtered at abattoirs. Therefore, it is concerning that *B. melitensis* that has been detected in sheep and goats in South Africa have now been identified in cattle. This information should be taken into consideration in the revision of the brucellosis testing scheme that is currently ongoing (DAFF, 2017). This abattoir survey provided useful baseline data on the prevalence of brucellosis in the tested animal population within a region or the country.

The vaccination status of the slaughtered animals in this study was unknown. Vaccination of 3–8 months heifers with *B. abortus* S19 is compulsory. However, the percentage of cattle vaccinated with S19 is unknown as the vaccine producer of S19 (Onderstepoort Biological Products) only indicate overall total vaccine sold. There was also a period when *B. abortus* S19 vaccine was unavailable (DAFF, 2017). There is no compulsory testing to monitor *B. melitensis* in sheep and goats in South Africa (DAFF, 2017). *Brucella melitensis* outbreaks reported in South Africa in sheep and goats have mostly been noticed only when human brucellosis was detected in individuals associated with sheep and goats (Emslie & Nel, 2002).

The detection of *B*.* abortus* bv 1 has been documented to be the common species and biotype infecting the cattle population in South Africa (Coetzer et al., 1994; Gradwell, 1977) and in other countries especially in the Southern African region and the Caribbean (Fosgate et al., 2002; Matope, Bhebhe, Muma, Skjerve, & Djønne, 2009; Muendo et al., 2012). The isolation of *B. melitensis* from cattle in this study is however significant because it is considered the first documentation of *B. melitensis* in the cattle population in South Africa (Kolo et al., 2018). The occurrence of *B. melitensis* in cattle in this study may be as a result of rearing cattle together with sheep, goats and wildlife on the same farm or sharing of grazing land with sheep or goats (Radostits et al., 2006; Verger, Garin‐Bastuji, Grayon, & Mahé, 1989). This has huge implication to infection of other cattle in the herd and most importantly spill over to other farms and other species around that geographical areas (Godfroid et al., 2014). *Brucella melitensis* bv 2 and 3 have never been isolated in livestock in the country. This has significant implications for brucellosis control in South Africa as the brucellosis scheme only focus on testing bovine high risk herds. As this study report *B. melitensis* bv 2 and 3 to be present in cattle, there is a high probability of its presence in the sheep and goat populations. *B. melitensis* has been isolated from sable antelope (DAFF, 2015) and biotyped as *B. melitensis* bv 3 (unpublished results). *Brucella melitensis* was isolated and reported in humans in the Western Cape province in 2015 (Wojno et al., 2016) and biotyped as *B. melitensis* bv 1 (van Heerden unpublished data). Furthermore, Caine et al. (2017) detected *B. melitensis* from the tissues and blood samples of sheep at abattoirs in the Eastern Cape province, but was not biotyped.

In our study, the detection of co‐infection by *B*. *melitensis* and *B. abortus* is being reported for the first time in cattle in South Africa. AMOS‐PCR indicated a mixed infection of *B. abortus* and *B*. *melitensis* (Figure 2) in an impure culture, but Bruce‐ladder is unable to differentiate a mixed infection of both organisms since it will only show the profile of *B. melitensis*. Mixed infection is plausible if morphological identical *Brucella* colonies of different species grow on the same plate. In future studies, the impure culture could be purified by picking multiple colonies resulting from the same animal and speciating the pure cultures using PCR followed by biotyping.

The possibility exists that a higher frequency of false positives and tests occurs when RBT is used relative to other tests which led to the recommendation that the RBT should not be used as a stand‐alone test to determine the seroprevalence of brucellosis (OIE, 2009). Since the current study was unable to confirm the vaccination status of the slaughter animals tested, the possibility of vaccine‐induced antibody production, unrelated to natural exposure to *Brucella* spp. by both tests cannot be ignored. However, molecular (Bruce‐ladder PCR) and isolation methods were used to differentiate vaccine strains from our isolates (Figure 3) and no vaccine strain was identified.

It is of diagnostic significance that *Brucella* DNA was detected using ITS‐PCR in 11 seronegative cattle along with 14 seropositive cattle, an overall prevalence of 12.5% (25/200). The ITS‐PCR (genus‐specific) is a good option as a screening tool for *Brucella* DNA as the PCR is capable of detecting very small amount of DNA in the tissue samples even if as little as 3.8 fg of *Brucella* DNA mixed with 450 ng of host DNA (Keid et al., 2007). This is significant because the application of PCR to detect *Brucella* DNA in animal tissues can be used to diagnose brucellosis in immune‐compromised animals that are unable to seroconvert following exposure and infection, and in animals where the *Brucella* organism, which is an intra‐cellular organism, is localized in the tissues, such that serology may not be able to diagnose the infection from the serum samples (Radostits et al., 2006). However, the ITS‐PCR sensitivity and specificity should be validated in South Africa especially to ensure the specificity of this PCR, as it could react with *Brucella*‐like organisms in this region that could not be tested in the initial validation by Keid et al. (2007). The PCR method used in our study has proven to be fast, safe and does not require specialized laboratory as required in the bacteriological methods. Based on the detection rate of 92.0% in the lymph node tissues in our study, we recommend the pooling of the lymph nodes initially to assay for *Brucella* DNA. The strategic application of bacteriology, serology and PCR was shown to result in increased detection frequency of brucellosis in cattle.

## 5 RECOMMENDATIONS

It is recommended that abattoir‐based study be conducted at provincial and national levels, to ascertain the frequency of brucellosis detection in all the nine provinces of the country. A serological testing strategy based on RBT and iELISA may be used in series considering the fact that CFT is very laborious to conduct, not as robust as other serological tests, such as the iELISA and difficult to standardize (OIE, 2009; Padilla, Nielsen, Ernesto, & Ling, 2010). This will provide baseline data for policy makers to proffer solutions and interventions to mitigate the risk of economic losses to livestock in the country and to mitigate the public health impact of the disease in the human population. It should be mandatory for livestock farmers to permit trace back to their farms if slaughtered animals from their farms are seropositive for brucellosis. This can be achieved by enforcing existing regulations.

## 6 LIMITATIONS

The use of serology alone can only be presumptive because other pathogens can cross‐react to yield false‐positive results. In our study, many of the RBT‐positive sera were negative on iELISA. Major inferences could not be made on serological data generated because of the unavailability of important variables such as the vaccination history. This is because some serological tests are unable to distinguish between antibodies produced in response to exposure to vaccination or natural exposure to *Brucella* spp. Although the data generated from abattoir studies may be invaluable to surveillance (passive and active), the animals sampled may not be representative of the animal population in the province, based on the sample size used and the movement of livestock from other provinces in the country to Gauteng province where the study was conducted from other provinces in the country. A trace back study to the farm sources from where abattoir‐tested seropositive cattle originated was not conducted due to the lack of cooperation by the farmers who were apprehensive of potential quarantine, with associated economic losses, due to anticipated enforcement of control measures.

## 7 CONCLUSIONS

In conclusion, this study has provided data that *B. melitensis* may be circulating in the cattle population in South Africa. *Brucella melitensis* bv 2 and 3 have never been isolated in the cattle population in the country**.** This suggests a potential risk to abattoir workers and to a lesser extent to the consumers of raw or under cooked meat and meat products. The study also provided a current data on the prevalence (isolation, serology and PCR) of brucellosis in slaughter cattle in Gauteng province, South Africa. The seroprevalence of brucellosis detected in slaughter cattle in this study emphasizes the importance of using abattoirs for passive and active surveillance of diseases of public health and economic importance. Our study also confirmed that as a diagnostic strategy, it is imperative to institute more than one diagnostic method or test for the diagnosis of brucellosis in animals.

## CONFLICT OF INTEREST

There is not conflict of interest with regards to this research.

## AUTHORS’ CONTRIBUTIONS

FK, carried out all of the experimental work and manuscript preparation. AA, FF and HvH played equal roles as the study leaders and in carrying out data analysis and manuscript preparation. BD, CK, IM, AG and AP were involved in the generation and analysis of the biochemical tests and amplicon data. All authors read and approved the final manuscript.

## ETHICAL STATEMENT

Permission to perform the research was granted regarding Section 20 Animal Diseases Act, 1984 (Act number 34 of 1984), by the Department of Agriculture Fisheries and Forestry, Reference number 12/11/1/1/6. Ethics approval was also granted by the University of Pretoria's Animal Ethics Committee, project number AEC12‐16.
